# Chronic Intermittent Hypoxia Triggers a Senescence-like Phenotype in Human White Preadipocytes

**DOI:** 10.1038/s41598-020-63761-7

**Published:** 2020-04-22

**Authors:** Katarzyna Polonis, Christiane Becari, C. Anwar A. Chahal, Yuebo Zhang, Alina M. Allen, Todd A. Kellogg, Virend K. Somers, Prachi Singh

**Affiliations:** 1 Department of Cardiovascular Medicine, Mayo Clinic, MN Rochester, USA; 2 Department of Surgery and Anatomy, Ribeirao Preto Medical School, Ribeirão Preto, SP Brazil; 3 Mayo Clinic Graduate School of Biomedical Sciences, MN Rochester, USA; 4 Division of Gastroenterology and Hepatology, Mayo Clinic, MN Rochester, USA; 5 Department of Surgery, Mayo Clinic, MN Rochester, USA; 6 Pennington Biomedical Research Center, LA Baton Rouge, USA

**Keywords:** Metabolic disorders, Risk factors

## Abstract

Obstructive sleep apnea (OSA) is a common sleep disorder associated with obesity. Emerging evidence suggest that OSA increases the risk of cardiovascular morbidity and mortality partly via accelerating the process of cellular aging. Thus, we sought to examine the effects of intermittent hypoxia (IH), a hallmark of OSA, on senescence in human white preadipocytes. We demonstrate that chronic IH is associated with an increased generation of mitochondrial reactive oxygen species along with increased prevalence of cells with nuclear localization of γH2AX & p16. A higher prevalence of cells positive for senescence-associated β-galactosidase activity was also evident with chronic IH exposure. Intervention with aspirin, atorvastatin or renin-angiotensin system (RAS) inhibitors effectively attenuated IH-mediated senescence-like phenotype. Importantly, the validity of *in vitro* findings was confirmed by examination of the subcutaneous abdominal adipose tissue which showed that OSA patients had a significantly higher percentage of cells with nuclear localization of γH2AX & p16 than non-OSA individuals (20.1 ± 10.8% vs. 10.3 ± 2.7%, *P*_*adjusted*_ < 0.001). Furthermore, the frequency of dual positive γH2AX & p16 nuclei in adipose tissue of OSA patients receiving statin, aspirin, and/or RAS inhibitors was comparable to non-OSA individuals. This study identifies chronic IH as a trigger of senescence-like phenotype in preadipocytes. Together, our data suggest that OSA may be considered as a senescence-related disorder.

## Introduction

Obstructive sleep apnea (OSA), a sleep breathing disorder, poses a significant worldwide public health problem associated with a higher cardiovascular and metabolic risk^1^. While it is well established that exposure to repetitive intermittent hypoxia (IH) during sleep contributes to OSA pathophysiology, the mechanisms by which the acute physiological disruptions translate into long-term health consequences are not completely understood. The importance of this issue is underscored by recent studies showing that continuous positive airway pressure (CPAP) therapy has been unable to effectively reduce cardiovascular events in OSA patients^2–4^. The inability of CPAP to diminish cardiometabolic risk suggests other mechanisms which do not readily reverse by elimination of IH, and may continue to mediate OSA-related pathogenesis, even after cessation of exposure to IH.

Senescence is a fundamental mechanism implicated in tissue dysfunction and aging processes, including chronic diseases such as diabetes, metabolic dysfunction and cardiovascular disease (CVD)^5–7^. In healthy tissue, senescent cells are eliminated by the immune system which drives tissue regeneration^8^; however, stress stimuli may corrupt this process and lead to increased accumulation of senescent cells. Increased senescence may limit tissue regenerative capacity and aggravate tissue dysfunction, presumably through intracellular signaling loops including senescence-associated secretory phenotype^9^. Therefore, senescence represents a self-propagating mechanism which can continue cellular damage even after the initial trigger has been eliminated.

The central metabolic role of adipose tissue is widely recognized in the pathogenesis of chronic inflammation, insulin resistance, metabolic syndrome and CVD^10^. Emerging evidence indicates that adipose tissue senescence may have profound clinical consequences for cardiovascular health including diabetes and age-related metabolic dysfunction^11^. Senescent preadipocytes are likely to limit the regenerative capacity of adipose tissue, alter adipocyte differentiation, lipid storage and free-fatty acid release, and therefore impact glucose and lipid homeostasis^12^. The functional significance of senescence is supported by animal studies showing that clearing senescent cells from different types of tissue, including adipose tissue, markedly improved age-related phenotypes^13^. The first evidence of the ability of senolytics, which selectively induce death of senescent cells, to alleviate physical dysfunction associated with age-related disorders in humans has also been recently presented^14^.

Despite a recognized role of adipose tissue in development of obesity and cardiometabolic disorders and growing evidence that sleep fragmentation and deprivation may promote cellular senescence^15,16^, little is known about the effects of OSA on adipose tissue and senescence^17^. Therefore, we sought to examine the ability of IH to induce a senescence-like phenotype in human white preadipocytes (HWPs), and to provide proof of concept that OSA is associated with higher levels of senescence related biomarkers in adipose tissue. Furthermore, we aimed to identify existing therapeutic options that may be used to attenuate OSA-related senescence-like phenotype. Our findings suggest that senescence-like phenotype, described by nuclear expression of γH2AX and p16, in the context of OSA is likely to be partly associated with IH-induced mitochondrial reactive oxygen species (ROS) generation.

## Results

### Intermittent hypoxia induces senescence in cultured preadipocytes

**Figure 1:**
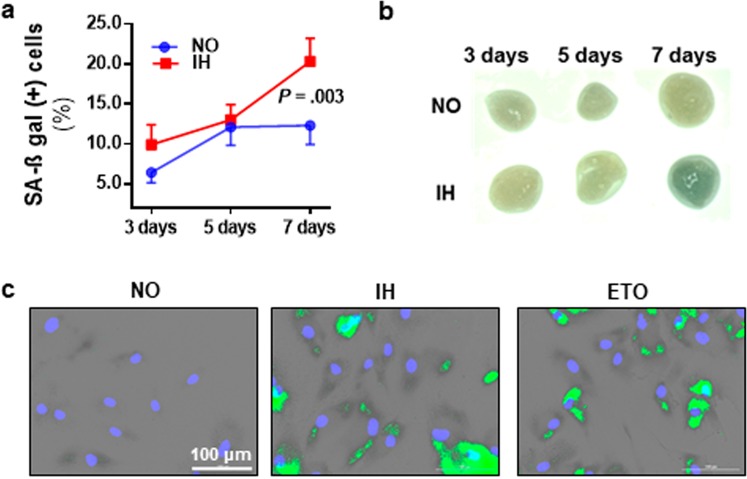
Intermittent hypoxia (IH) induces senescence in cultured preadipocytes. Exposure to 7 days of IH was associated with a higher prevalence of senescence associated β-galactosidase (SA-β-gal) positive preadipocytes (n = 6 independent experiments) (**a**). Compared to tissue grown in continuous normoxia (NO), chronic IH exposure was also associated with stronger green coloration of SA-β-gal staining in subcutaneous adipose tissue explants (*ex vivo*) (**b**). Representative images of SA-β-gal staining in cells exposed to 7 days of IH (**c**). Green marks indicate SA-β-gal positive (senescent) cells; Etoposide (ETO) treatment served as a positive control. Data are presented as mean ± SEM. *P*-values determined by one-tailed paired t-test compared to the NO control.

**Figure 2:**
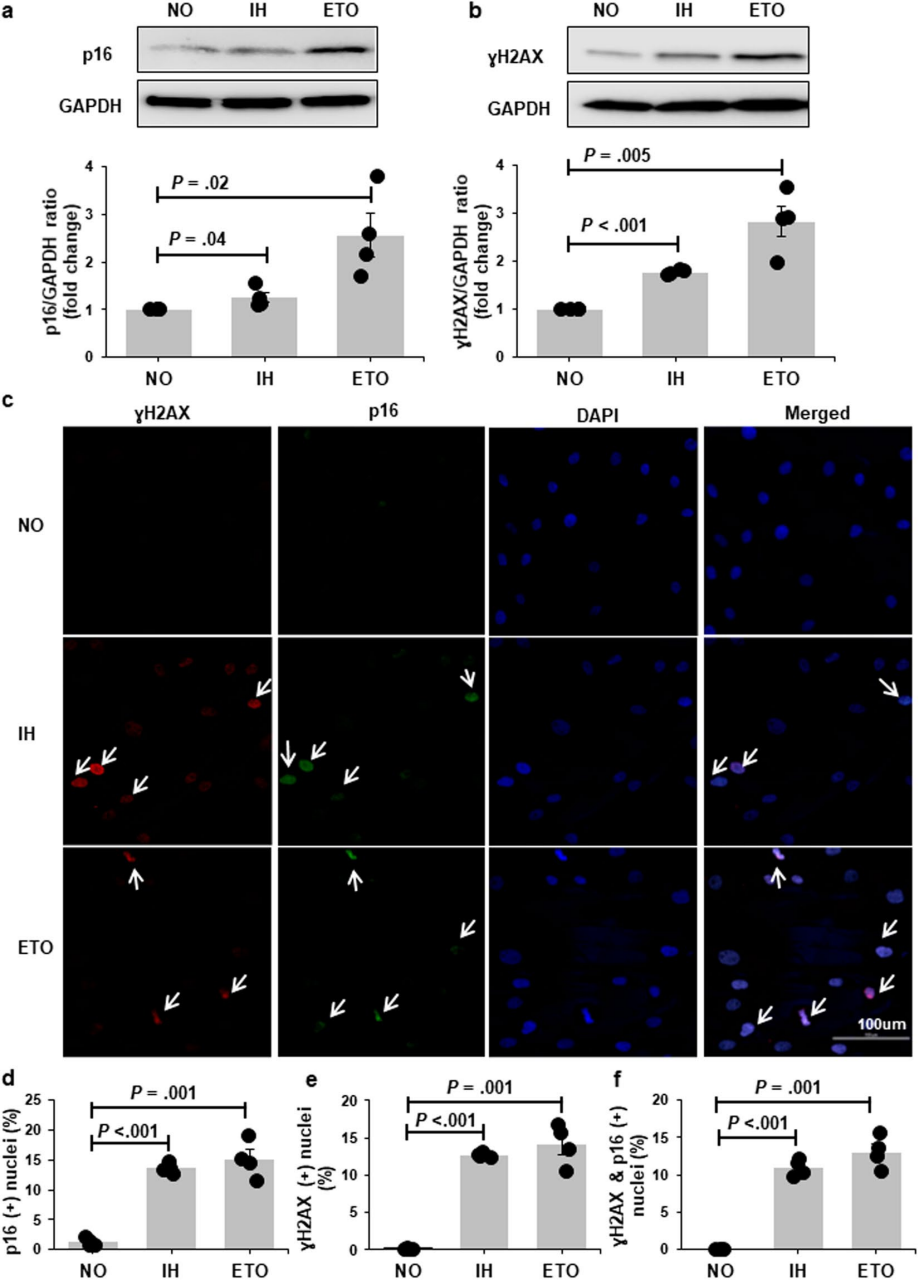
P16 and γH2AX expression is increased with chronic exposure to intermittent hypoxia (IH). Representative Western bolts and graphs showing upregulation of p16 (**a**) and γH2AX (**b**) protein expression with IH treatment. Representative confocal images showing increased nuclear localization of p16 (green) and γH2AX (red) in cells exposed to IH (**c**). Nuclei are counterstained blue (DAPI). White arrows indicate positive nuclei. Quantitation of cells positive for nuclear p16 (**d**), γH2AX (**e**) and p16&γH2AX (**f**) in preadipocytes grown in continuous normoxia (NO) versus cells grown with intermittent exposure to hypoxia (n = 4 independent experiments). Cells treated with etopside (ETO) were used as positive control. Data are presented as mean ± SEM. *P*-values determined by one-tailed paired t-test compared to the NO control.

**Figure 3:**
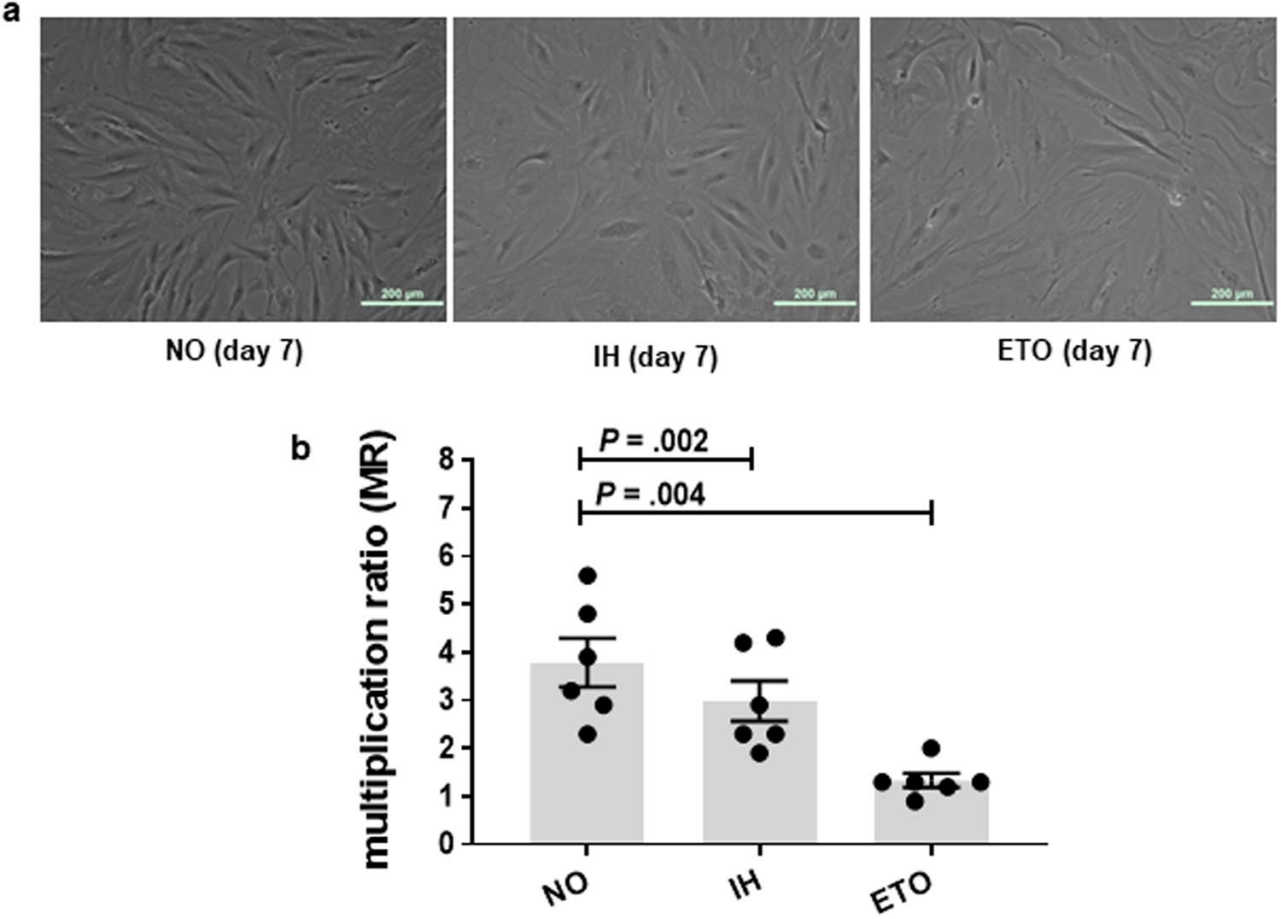
Intermittent hypoxia (IH) alters preadipocyte phenotype and replicative capacity. Chronic IH was associated with a characteristic set of phenotypic changes such as flattened and enlarged cells (**a**) and a reduced multiplication ratio (MR) (**b**). Etoposide (ETO) was used as a positive control. Data are presented as mean ± SEM. *P*-values determined by one-tailed paired t-test compared to the normoxia (NO) control (n = 6 independent experiments).

We examined the effect of chronic IH on senescence by exposing HWPs to 3, 5 and 7 days of IH or continuous normoxia. The percentage of senescence associated β-galactosidase (SA-β-gal) positive cells were significantly higher after 7 days of IH exposure as compared to normoxia (IH 20.3 ± 2.9% vs. NO 12.3 ± 2.4%, *P* = 0.003) (Fig. 1a,c). Similarly, we found stronger β-gal staining, indicating increased senescence, in subcutaneous adipose tissue explant (*ex vivo*) exposed to 7 days of IH treatment (Fig. 1b). Compared to normoxia, chronic IH was also associated with increased protein expression of p16 and γH2AX (Fig. 2a,b) along with an increase in the prevalence of cells with nuclear localization of p16, γH2AX and both γH2AX & p16 (Fig. 2c–f). Furthermore, chronic IH treatment was accompanied by a characteristic set of phenotypic changes such as enlarged and flattened cells and significantly lower multiplication ratio (*P* = 0.002) (Fig. 3a,b).

**Figure 4:**
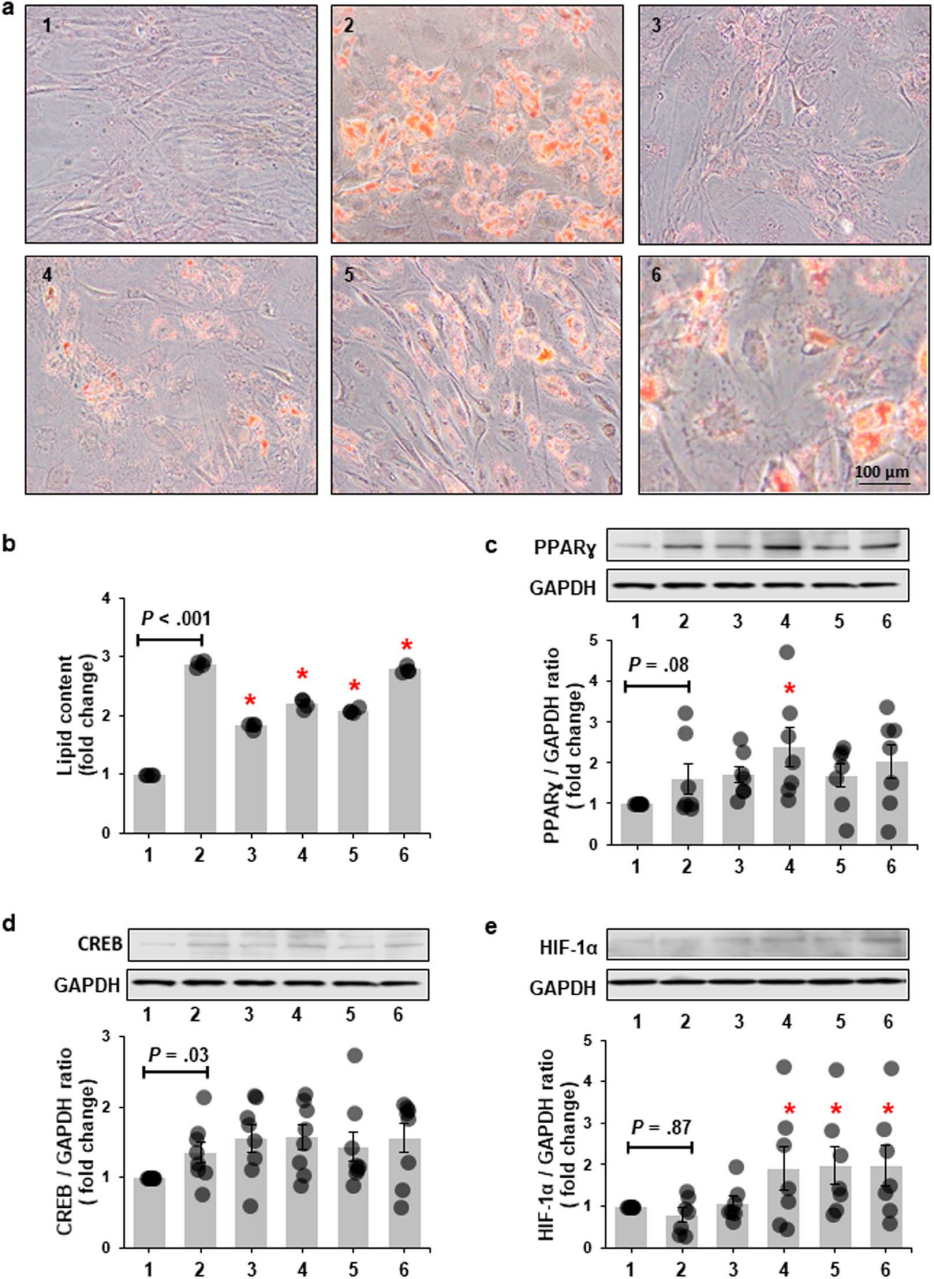
Exposure of preadipocytes to chronic intermittent hypoxia (IH) attenuates lipid accumulation during differentiation. Representative images of Oil-Red-O stained preadipocytes differentiated for 6 days (**a**). Quantitation of lipid content accumulated in differentiated cells (**b**) (n = 4 independent experiments). Compared to preadipocytes grown in continuous normoxia (NO), preadipocytes exposed to IH show less lipid accumulation at day 6 of differentiation. Representative Western Blots and graphs showing protein expression of peroxisome proliferator-activated receptor γ (PPARγ) (**c**) (n = 7 independent experiments), cAMP response element-binding (CREB) (**d**) (n = 8 independent experiments), and hypoxia-inducible factor-1α (HIF-1α) (**e**) (n = 7 independent experiments) in 6 day differentiated preadipocytes. IH elevated expression of PPARγ and CREB even in absence of exposure to differentiating media. HIF-1α expression was elevated in preadipocytes exposed to IH prior to treatment with differentiating media. **1**: NO; **2**: NO + differentiation in NO; **3**: IH; **4**: IH + differentiation in NO; **5**: IH + differentiation in IH; **6**: etopside + differentiation in NO. Data are presented as mean ± SEM. *P*-values determined by one-tailed paired t-test compared to the cells grown and differentiated in continuous NO (2). *P < 0.05. Etopside was used as a positive control. The same blot was used for the representative images of PPARγ and CREB. Therefore, the image for GAPDH protein is also the same.

We also determined the effects of chronic IH on adipogenesis. We show that prior chronic exposure of HWPs to IH attenuated lipid accumulation during differentiation (Fig. 4a,b). Chronic IH exposure also increased the expression of known adipogenic transcription factors such as peroxisome proliferator-activated receptor γ (PPARɣ) and cAMP response element-binding (CREB) which associates with lipid accumulation even in absence of exposure to differentiating media (Fig. 4c,d). Pretreated HWPs in presence of differentiating media showed increased expression of hypoxia-inducible factor 1α (HIF-1α) as well (Fig. 4e).

### Mitochondrial oxidative stress and DNA damage accompany IH-induced preadipocyte senescence-like phenotype

**Figure 5:**
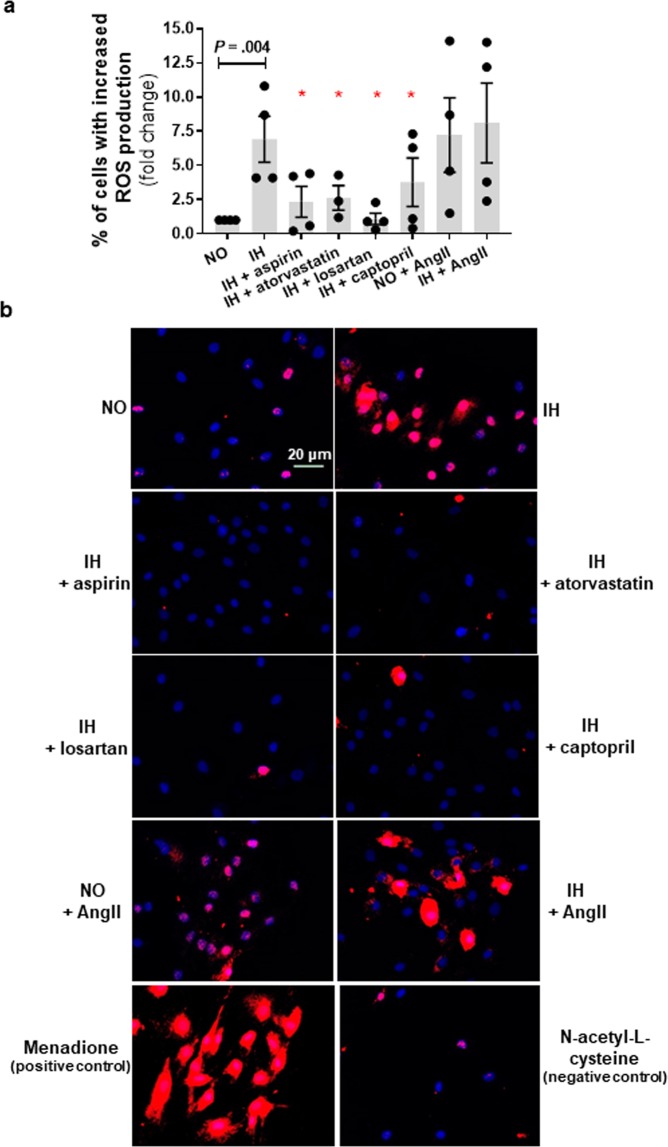
Mitochondrial reactive species generated during chronic intermittent hypoxia (IH) exposure in preadipocytes is attenuated in presence of aspirin, atorvastatin and drugs targeting renin-angiotensin system. Intervention with aspirin, atorvastatin, angiotensin II type-1 receptor antagonist (losartan), and angiotensin converting enzyme inhibitor (captopril) reduced IH-induced mitochondrial reactive oxygen species generation while angiotensinII (AngII) increased mitochondrial reactive oxygen species generation as indicated by MitoSox staining (**a**) Representative images of MitoSox staining with red color indicating increased ROS (**b**). Cells treated with menadione (150 µM) served as a positive control and cells treated with N-acetyl-L-cysteine (50 µM) served as a negative control. Data are presented as mean ± SEM, *P < 0.05 determined by one-tailed paired t-test (n = 3–5 independent experiments) as compared to IH. NO: normoxia.

**Figure 6:**
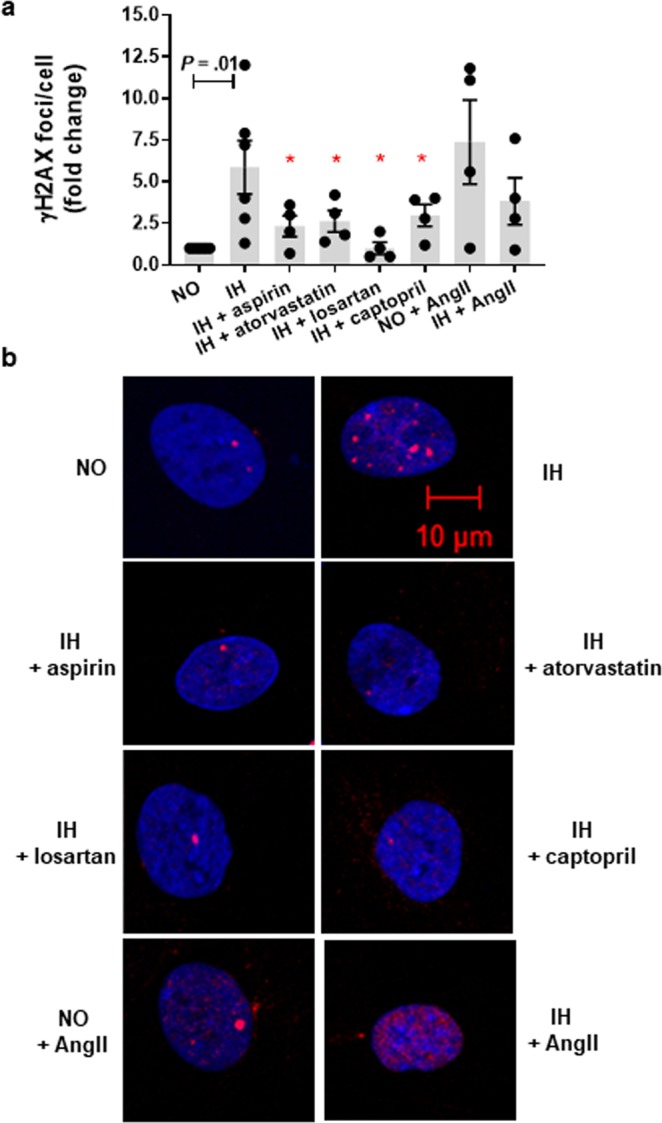
Increases in nuclear H2AX foci during intermittent hypoxia (IH) is reduced by concomitant exposure to aspirin, atorvastatin and drugs targeting renin-angiotensin system. Treatment with aspirin, atorvastatin, angiotensin II type-1 receptor antagonist (losartan), and angiotensin converting enzyme inhibitor (captopril) was associated with reduced IH-induced DNA damage as indicated by H2AX phosphorylation (**a**). Angiotensin II (AngII) treatment resulted in increased DNA damage in both normoxia (NO) as well as IH. Representative confocal images of immunofluorescence staining with red color indicating phosphorylated H2AX foci (**b**). Data are presented as mean ± SEM, *P < 0.05 determined by one-tailed paired t-test (n = 3–5 independent experiments) as compared to IH.

We demonstrated that chronic IH exposure was associated with mitochondrial oxidative stress as determined by prevalence of MitoSOX positive cells (Fig. 5) and increased H2AX phosphorylation (Fig. 6). In the IH group, there was 6.9 (±1.7) fold increase in cells with elevated mitochondrial oxidative stress and their nuclei had on average 5.9 (±1.6) fold increase in γH2AX foci/cell as compared to normoxia.

### Drugs with anti-oxidative properties attenuate IH-induced senescence

**Figure 7:**
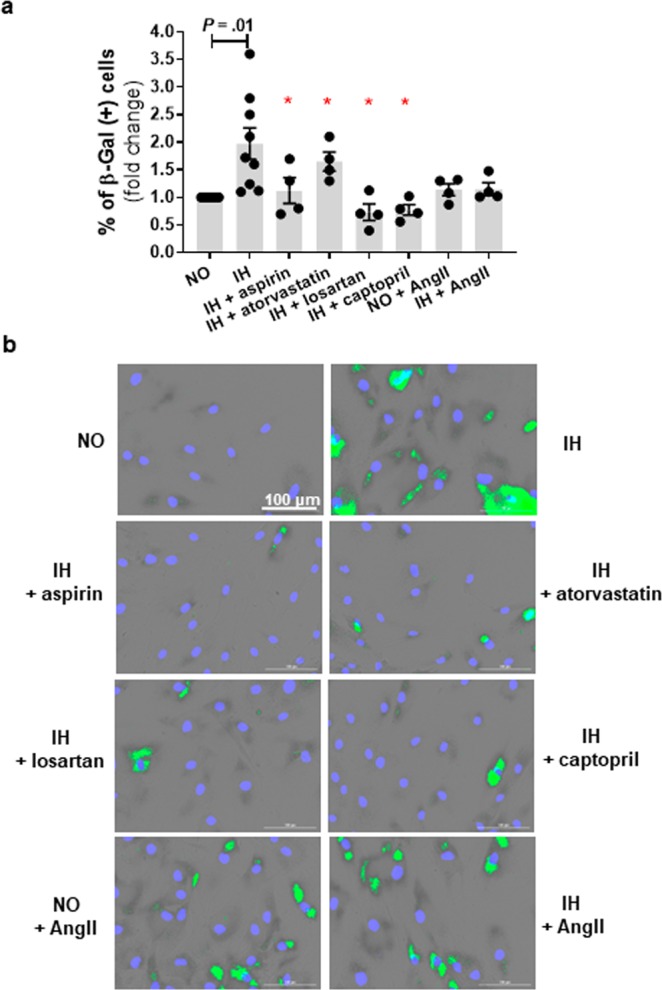
Intermittent hypoxia (IH)-induced senescence is attenuated by treatment with aspirin, atorvastatin, and renin-angiotensin system targeted drugs. IH mediated increased prevalence of SA-β-gal positive cells was attenuated by concomitant treatment with aspirin, atorvastatin, angiotensin II type-1 receptor antagonist (losartan), and angiotensin converting enzyme inhibitor (captopril) (**a**). Representative images of SA-β-gal staining with green marks indicating senescent cells (**b**). Data are presented as mean ± SEM, *P < 0.05 determined by one-tailed paired t-test (n = 3–5 independent experiments) as compared to IH. NO: normoxia; AngII: angiotensinII.

Statins and aspirin are commonly used in the primary prevention of CVD in at-risk populations while renin-angiotensin system (RAS) inhibitors are commonly used in the treatment of hypertension, which is highly prevalent in OSA population. One of beneficial effects displayed by aspirin and statin is linked with protecting against oxidative stress and oxidative damage^18^. Oxidative stress and mitochondrial dysfunction are also proposed mechanisms linking RAS to cardiometabolic disturbances^19^. Therefore, we examined the effects of these drugs on IH mediated induction of senescence-like phenotype. We demonstrate that aspirin and atorvastatin treatment reduced the levels of IH-induced mitochondrial oxidative stress (*P* = 0.02 and *P* = 0.02, respectively) in cultured preadipocytes (Fig. 5). Consequent reduction of H2AX phosphorylation (*P* = 0.03 and *P* = 0.02, respectively) and prevalence of senescent (SA-β-gal positive) cells were also evident (*P* = 0.003 and *P* = 0.006, respectively) (Figs. 6 and 7). The level of mitochondrial oxidative stress, H2AX phosphorylation and SA-β-gal positive HWPs in the aspirin group was similar to normoxia (*P* = 0.43, *P* = 0.26, *P* = 0.46, respectively). Atorvastatin treatment also reduced mitochondrial oxidative stress and SA-β-gal positive cells to levels comparable with normoxia (*P* = 0.06 and *P* = 0.27), even though H2AX phosphorylation was still significantly higher than in normoxia (*P* = 0.04).

Next, we showed that treatment with angiotensin II (AngII), a main peptide effector of RAS, was associated with a higher mitochondrial oxidative stress (*P* = 0.02, Fig. 5), H2AX phosphorylation (*P* = 0.06, Fig. 6), and SA-β-gal activity (*P* = 0.05, Fig. 7) in HWPs grown in continuous normoxia. Importantly, the AngII mediated induction of senescence (SA-β-gal positive cells) was comparable to the IH group (*P* > 0.05). There was no additive effect of AngII and IH treatment on mitochondrial oxidative stress, and preadipocyte senescence (*P* > 0.05). Also, we demonstrated that losartan (AT1R antagonist) and captopril (ACE inhibitor) reduced IH-mediated mitochondrial oxidative stress (*P* = 0.009 and *P* = 0.04, respectively, Fig. 5), H2AX phosphorylation (*P* = 0.02 and *P* = 0.03, respectively, Fig. 6), and SA-β-gal activity (*P* = 0.03 and *P* = 0.002, respectively, Fig. 7). The prevalence of SA-β-gal positive cells in the losartan and captopril treatment group was similar to the normoxia group (*P* = 0.15 and *P* = 0.12).

### OSA patients have higher cellular damage in subcutaneous adipose tissue

*In vitro* experiments suggested that chronic IH exposure in OSA patients may contribute to cellular damage and senescence in adipose tissue. To determine the clinical relevance of *in vitro* findings, we examined nuclear localization of γH2AX and p16 (a surrogate marker of senescence that correlates with results of SA-β-gal staining) in abdominal adipose tissue of non-OSA and OSA subjects. We also investigated adipose tissue from OSA patients receiving medication targeting oxidative stress (aspirin/statin) and/or RAS pathways. Compared to OSA patients, the non-OSA individuals were healthier, younger, and had a lower BMI (Table 1).

**Table 1:** Characteristics of the subjects according to CPAP usage and medications.

	non-OSA n = 14	CPAP(−)		CPAP(+)	
		OSA-med(−) n = 11	OSA-med(+) n = 5	OSA-med(−) n = 11	OSA-med(+) n = 9
Age, *years*	30.0 ± 1.5^a^	44.0 ± 3.5^b^	56.6 ± 4.5^c^	43.8 ± 3.1^b^	55.3 ± 3.1^c^
BMI, *kg/m*^2^	29.1 ± 2.1^a^	34.0 ± 3.2^a^	33.6 ± 2.9^a^	48.1 ± 2.0^b^	45.1 ± 1.7^b^
Male sex, *n (*%*)*	6 (43%)	7 (64%)	3 (60%)	2 (18%)	1 (11%)
Dyslipidemia, *n (%)*	0 (0%)	0 (%)	3 (60%)	0 (0%)	5 (56%)
Hypertension, *n (%)*	0 (0%)	2 (12%)	3 (60%)	5 (45%)	7 (78%)
γH2AX & p16 (+) nuclei, *%*	10.3 ± 0.7	26.2 ± 3.4	12.2 ± 4.9	23.0 ± 2.5	15.6 ± 2.4

Data are presented as mean ± SEM and the count with percentage (%). OSA – obstructive sleep apnea, BMI – body mass index, CPAP – continuous positive airway pressure, med – refers to medication including renin-angiotensin inhibitors, statins and/or aspirin, (+/−) – present or absent. Levels not connected by the same letter are significantly different (*P *< 0.05).

**Figure 8:**
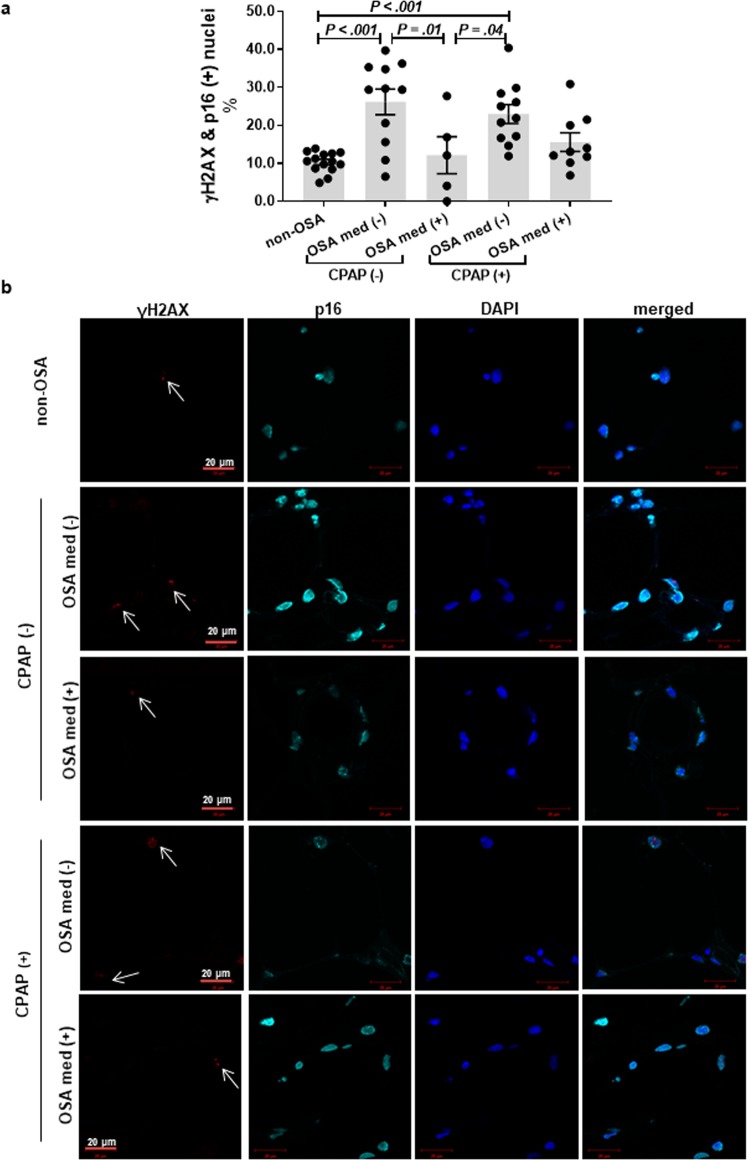
Adipose tissue of OSA patients show increased expression of nuclear p16 and γH2AX. OSA was associated with a higher prevalence of cells with senescence-like phenotype in subcutaneous adipose tissue as determined by nuclear localization of both γH2AX and p16 **(a)**. The prevalence of dual positive nuclei was not attenuated by CPAP usage but was altered by concomitant use of medications. Representative images of immunofluorescence staining with white arrows indicating γH2AX positive nuclei **(b)**. OSA – obstructive sleep apnea, CPAP – continuous positive airway pressure, med – statins, aspirin and/or renin-angiotensin system inhibitors, (+/−) – present or absent. Data are presented as mean ± SEM. *P*-values determined by linear regression model adjusted for age and BMI.

Overall, adipose tissue of OSA subjects had higher prevalence of γH2AX & p16 positive nuclei as compared to non-OSA subjects after adjusting for age and BMI (20.8 ± 10.4% vs. 10.3 ± 2.7%, *P* < 0.001) (Fig. 8). The frequency of dual positive γH2AX & p16 nuclei in the adipose tissue was also significantly higher in OSA patients receiving CPAP therapy (but no medications) as compared to non-OSA subjects (*P* < 0.001). Importantly, OSA subjects receiving statins and/or RAS inhibitors had a significantly lower percentage of γH2AX & p16 (+) nuclei in adipose tissue than OSA subjects with no such treatment (12.2 ± 4.9% vs. 26.2 ± 3.4%, *P* = 0.01), and this was comparable to the non-OSA group (*P* = 0.18). This is a particularly interesting observation as the OSA medication (+) group was on average 27 years older than non-OSA group (56.6 ± 4.5 vs. 30.0 ± 1.5 years) (Fig. 8).

## Discussion

The main finding of this study is that chronic IH induces a senescence-like phenotype in primary human preadipocytes which includes elevated SA-β-gal activity, increased nuclear localization of γH2AX & p16 along with accompanied increases in mitochondrial ROS. These *in vitro* findings are consistent with higher prevalence of cells with γH2AX & p16 nuclear localization in subcutaneous abdominal adipose tissue of OSA patients. To our knowledge, this is the first study to provide evidence that OSA may be considered as a senescence-related disorder. Our data suggest that senescence is a potential cellular mechanism which may contribute to OSA-related pathophysiology and indicate viable therapeutic options to limit senescence-like characteristics in OSA patients.

DNA damage is a well-recognized central driver for establishing both replicative and premature (stress-induced) cellular senescence in response to oxidative stress. Therefore, any disruption of biological systems that increases intracellular ROS levels and/or reduce capacity of DNA repair mechanisms would be expected to induce cellular senescence^20,21^. OSA, which is characterized by IH, may be considered a disruptor of oxidative balance by inducing mitochondrial dysfunction leading to increased mitochondrial ROS production and consequent DNA damage^22^. Oxidative stress and mitochondrial dysfunction is also one of the proposed mechanisms underlying OSA pathophysiology^23^, linking activated RAS to cardiometabolic disturbances^19^, and finally implicated in the senescence induction^24,25^. Thus, our focus to identify potential beneficial therapeutic interventions was on drugs with anti-oxidative properties such as statin and aspirin and those targeting activated RAS such as an angiotensin converting enzyme inhibitor (ACE) and angiotensin receptor blockers^18^.

AngII has been demonstrated previously to have pro-senescence actions in vascular endothelial and smooth muscle cells^26,27^. In this study, we show that AngII treatment was associated with increased mitochondrial ROS production, DNA damage, and higher percentage of SA-β-gal positive cells, while inhibiting AngII generating pathways or inhibiting cellular actions of AngII attenuated IH-associated senescence-like phenotype. This observation supports the contributing role of AngII in triggering mitochondrial ROS and senescence (as defined by SA-β-gal positive cells) in conditions of IH. Previous studies have also shown attenuation of AngII-mediated senescence by antagonizing AT1R, down regulating AT1R signaling or preventing AngII generation^26,28–31^.

Our results also confirm pleiotropic effects of statin and aspirin such as reducing stress-induced premature senescence. Similar anti-senescent effects of statins and aspirin have been previously reported in endothelial progenitor, endothelial, and vascular smooth muscle cells^32–35^. These anti-senescent effects may be linked, at least partially, to reducing oxidative stress, DNA damage, and preventing hyper-responsiveness of AT1R to AngII stimulation^36,37^. The translational importance of our *in vitro* studies are corroborated by *in vivo* findings showing that adipose tissue of OSA patients taking medications such as statins, aspirin and/or RAS inhibitors has lower prevalence cells with senescence-like phenotype.

Identification of the IH-RAS-senescence axis may have significant clinical implications because RAS operates locally in various tissues mediating both autocrine and paracrine mechanisms. Local RAS may exacerbate the effects of circulating RAS and/or work independently to induce different cellular responses, which potentially include accumulation of senescent cells^38–40^. Therefore, this mechanism may be responsible for not only adipose tissue dysfunction, but may also contribute to other pathophysiology related to adverse outcomes in OSA patients. Moreover, the presence of localized RAS in different types of tissue may underlie the pleiotropic effects of RAS inhibitors in improved glycemic control and reduced risk of developing diabetes as seen in high cardiovascular risk populations^41–44^.

In a broader context, our findings provide a possible explanation for why recent clinical trials showed no significant effects of CPAP monotherapy in reducing cardiovascular risk^2–4^. CPAP eliminates the nightly exposure to IH; however, pre-existing senescent/senescent-like cells, resulting from IH exposure *prior* to initiation of CPAP treatment, may have a major effect on surrounding cells through direct cell-cell contacts and/or senescence-associated exosomes and macrovesicles^45^. It is through these interactions that preexisting senescent cells may likely promote nearby cells to become senescent which may conceivably contribute to continued tissue dysfunction and cardiometabolic risk. In other words, removing the IH-trigger alone may not be sufficient to improve tissue function. Consistent with the above hypothesis, we show that CPAP-therapy alone was not associated with lower adipose tissue cellular damage, while the use of RAS inhibitors, aspirin and/or statins (with- or without CPAP) was associated with overall reduced levels of adipose tissue senescence-related phenotype. This is consistent with suggestions that OSA patients who receive both CPAP and hypertensive treatment may have better cardiovascular outcomes^4^.

There are some inherent limitations to the study. The process of senescence is hard to characterize partly due to the underlying heterogeneity for markers which may not be exclusively expressed in senescent cells. To overcome this limitation we used multiple markers to characterize the senescence-related phenotype in the primary cultured HWPs after IH exposure. However, due to limitation of adipose tissue availability, we were confined to only using γH2AX and p16 staining as a surrogate senescence-related biomarker in our study subjects. Furthermore, the OSA and non-OSA groups were not matched for age and BMI. However, OSA patients receiving statins, aspirin and/or RAS inhibitors had similar adipose cellular damage compared to non-OSA individuals despite being significantly older. Also, the association between OSA and senescence-markers was not adjusted for OSA severity because this information was not available for all study participants. Last, we did not characterize detailed molecular mechanisms underlying IH-mediated senescence as this study mainly aimed to explore IH as a senescence trigger. Studies related to detailed molecular mechanisms facilitating development of senescence are being undertaken including non-mitochondrial mechanisms for increased ROS generation.

The pathophysiological significance of increased senescence in adipose tissue in the context of OSA still needs to be determined. Nevertheless, our findings have provided a robust basis for ongoing clinical trial to longitudinally examine the effect of CPAP treatment on adipose tissue cellular damage in the presence and absence of statin therapy (ClinicalTrials.gov, NCT03308578).

In summary, this study identifies OSA as a senescence-related disorder and provides a link between IH, RAS activation, and senescence-like phenotype in adipose tissue. Our findings offer a framework for identification of therapeutic strategies to prevent OSA-induced senescence (as an adjunct to CPAP therapy) and reduce cardiometabolic burden in OSA patients.

## Methods

### Cell culture, intermittent hypoxia model and reagents

All *in vitro* experiments were performed using commercially available primary human white preadipocytes (HWPs) isolated from abdominal adipose tissue of healthy individuals (ZenBio Inc., North Carolina). HWPs were grown in complete medium (PM-1, ZenBio, Inc., North Carolina) and used for experiments in 3-5 passage with a 50–60% confluence. A previously developed *in vitro* intermittent hypoxia model, mimicking a molecular signature seen in OSA patients, was used to examine the effect of IH on senescence^46,47^. Cells were exposed to either continuous normoxia (NO) condition (21% O_2_) or to 9 cycles of intermittent hypoxia (IH) (30 min of 21% O_2_ followed by 30 min of 0.1% O_2_) per day for up to 7 consecutive days in complete media. Brief exposure of the cells to 0.1% oxygen in presence of complete media leads to decrease in oxygen in the media by approximately 20% which is comparable to what may be experienced by OSA patients. Experiments were performed using the OxyCycler C42 (BioSpherix, New York) that allows precise % O_2_ and % CO_2_ profiling. For experiments evaluating the effects of chronic IH pre-exposure on adipogenesis, preadipocytes were grown in adipocyte differentiating medium for 6 days (DM-2, ZenBio Inc).

In the senescence attenuation experiments, cells were exposed to IH in presence of drugs to obtain maximal effect based on prior studies: AngII receptor type 1 (AT1R) antagonist (losartan, 1 µM, Sigma #1370462)^48,49^, angiotensin-converting enzyme (ACE) inhibitor (captopril, 1 µM, Sigma #C4042)^50,51^, statin (atorvastatin, 5 µM, Sigma #PZ001)^52^, and aspirin (1 µM, Sigma #A53376)^53,54^ for 7 days of IH. Cells grown in IH and normoxia were also treated with angiotensin II (Ang II, 100 nM, Sigma #A9525) for 7 days^49,55^.

Multiplication ratio of HWPs was determined in experimental conditions as indicative of capacity of cells to divide in cultured conditions. Cells were seeded at ~6,000 cells per cm^2^ and allowed to attach to a plate surface overnight, and then subjected to 7 days of IH and NO. After the treatment cells were harvested using a trypsin-EDTA solution and counted with a hemocytometer. Multiplication ratio (MR) was calculated as a ratio of the total number of cells harvested to the total number of cells seeded^56^. Cells treated with etoposide (12.5 µM, Cell Signaling Technology, #2200) for 72 h and allowed to recover for 4 days were used a positive control.

### Senescence associated β-galactosidase staining

The prevalence of senescence was determined by SA-β-galactosidase staining that is a gold standard to assess cellular senescence^57^. Staining was performed using the Senescence β-galactosidase Staining Kit (Cell Signaling Technology, #9860) following the manufacture’s standard protocol. Cells treated with etoposide (12.5 µM, Cell Signaling Technology, #2200) for 72 h and allowed to recover for 4 days were used a positive control were applicable.

Images were taken at 20x magnification with the Axiovert 200 M inverted microscope with AxioVision software (Zeiss) (4 images per each time point and/or treatment group, on average 96 cells scored) or acquired with Cytation 5 (Cell Imaging Multi-Mode Reader, BioTek®) (scan area: 2.9 mm^2^, on average 251 cells scored). SA-β-gal staining for a given experiment was performed at the same day to ensure identical sample processing, including an image acquisition. Scoring was performed with ImageJ software^58^ or Imager Software available for Cytation 5. Thresholding to identify senescent cells was set for a positive control (etoposide treatment) and then applied to every image to be compared to ensure objectivity^59^. Cells considered SA-β-gal positive were indicated by green marking, scored and presented as a percentage of SA-β-gal positive cells of the total cell population for a given treatment group.

### Immunofluorescence (IF) analysis

For *in vitro* experiments, post-treatment cells were fixed with 4% paraformaldehyde for 15 min and rinsed with PBS three times. After rinsing, cells were blocked with 2% goat serum for 60 min, washed and incubated overnight at 4 °C with primary antibody (p16: MA5-17142, 1:500 dilution, Invitrogen; γH2AX: PA5-28778, 1:500 dilution, Invitrogen). Cells were then washed with PBS and incubated with appropriate secondary antibodies (Goat anti-Rabbit IgG (H + L) Cross-Adsorbed Secondary Antibody, Texas Red, T-2767, 1:3000 dilution, Invitrogen; Goat anti-Mouse IgG (H + L) Secondary Antibody, FITC, 62-6511, 1:3000 dilution, Invitrogen) for 1 h in the dark, washed, and mounted with antifade mounting medium containing DAPI (Vectashield, H-1200, Vector Laboratory).

For paraffin-embedded tissue, the sections were deparaffinized, rehydrated, and antigens retrieved by incubation in 10 mM sodium citrate buffer (pH 6.0) at 95 °C for 20 min. Samples were blocked with 3% BSA/0.1% Tween 20 in DPBS for 4 hours at room temperature and incubated with anti-p16 antibody (ab54210, dilution 1:250) and anti-γH2AX antibody (ab11174, dilution 1:500) overnight at 4 °C. Next, samples were incubated with secondary antibodies (Alexa Flour 568 #A11019 and Cy5 #A10523 Invitrogen^TM^, dilution 1:1,000) for 1 hr and mounted with ProLong® Gold Antifade Mountant (Invitrogen™) containing DAPI.

Confocal images were acquired with Zeiss LSM780 microscope at 40x magnification. A tissue cross-section from obese and OSA patients were considered as a positive control. Nuclei positive for p16 and γH2AX were manually scored and expressed as a percentage of total number of cells. Additionally, in the *in vitro* senescence attenuation experiments the average number of γH2AX foci per nuclei was scored precisely by applying a threshold to each image using *Image J*.

### Quantification of mitochondrial oxidative stress

MitoSOX Red (#M36008, Invitrogen^TM^) was used to assess mitochondrial reactive oxygen species generation in preadipocytes cultured under normoxia and chronic IH conditions. Cells were loaded with 5 µM MitoSox Red for 20 min in the last cycle of intermittent hypoxia in HBSS/CA/Mg buffer and then counterstained with Hoechst 33342 (NucBlue™ Live ReadyProbes™ Reagent, Invitrogen™) to visualize nuclei. Cells treated with menadione (150 µM, Sigma-Aldrich) were positive control and cells treated with N-acetyl-L-cysteine (50 µM, Sigma-Aldrich) were negative control. Images were analyzed with *Image J* to identify cells with a positive signal above a set minimum threshold determined by examining positive and negative controls. Cells with indication of increased oxidative stress were scored and expressed as a percentage of positive cells to the total count of cells.

### Western blot analysis

Semi-quantification of intracellular proteins of interest was undertaken using standard Western Blot analysis. Post-treatment, cells were lysed (Pierce RIPA lysis and extraction buffer, 89901, Thermo Scientific) and 30 µg of total cell protein extract was loaded onto 12% SDS PAGE gel for electrophoresis and transferred to PVDF membrane. The membranes were then blocked with 5% milk (BioRad, #1706404XTU) in Tris buffered saline containing 0.2% Tween 20 for 1 hour, and incubated overnight with primary antibodies (PPARγ: sc-7273, 1:800 dilution, Santa Cruz Biotechnology Inc; CREB: ab31387, 1:800 dilution, Abcam; HIF-1α: #3716, 1:500 dilution, Cell Signaling Technology Inc; p16: #80772, 1:800 dilution, Cell Signaling; γH2AX:  #7631, 1:800, Cell Signaling; GAPDH: #2118, 1:2000 dilution, Cell Signaling Inc). After incubation with primary antibodies, the membranes were washed and incubated with appropriate secondary HRP conjugated antibody for 1 hour (Anti-mouse HRP: #7076s, 1:2000 dilution; Anti-rabbit HRP: #7074s, 1:2000 dilution, Cell Signaling Technology Inc). The membranes were developed with HRP substrate (Immoblion Forte Western HRP substrate, WBLUF0100, Millipore Sigma) and images were acquired with LI-COR Odyssey Fc imaging system (LI-COR Bioscience). Quantification of proteins of interest was done using Image Studio Version 4.0 (LI-COR Bioscience) and analyzed as ratio to GAPDH (endogenous control).

### Lipid accumulation analysis

After 6 days of differentiation, cells were washed with phosphate buffered saline (PBS) and fixed using 4% paraformaldehyde. After fixation, cells were washed and stained for neutral lipids using Oil Red O stain (0.3% in 60% isopropanol; O0625, Sigma Aldrich) for 30 min followed by washing with PBS to remove excess stain. Images of stained cells were acquired and quantification of lipid accumulation was done by extracting Oil Red O stain with 100% isopropanol and measuring absorbance at 490 nm.

### Human subjects

The study was approved by the Mayo Clinic Institutional Review Board (IRB) and written informed consent was obtained from all subjects. All procedures were followed per institutional guidelines. Banked adipose tissue of OSA and non-OSA subjects from our previous Mayo Clinic IRB approved studies (n = 50) were used to examine the prevalence of senescence. Subjects from whom the tissue samples were used for this study included individuals undergoing bariatric surgery and those participating in sleep-related research studies in our laboratory. Clinical and research records were reviewed to obtain demographics and clinical information such as chronic conditions and medication usage (Table 1**)**. Subjects with a clinical diagnosis of diabetes and/or cardiovascular disease were excluded. Presence of hypertension and dyslipidemia were confirmed based on clinical diagnosis and/or a list of medications. Sleep apnea was identified by reviewing records for overnight oximetry, polysomnography or clinical notes related to the sleep apnea treatment.

Considering the limitation related to tissue availability, a senescence phenotype was determined based on the nuclear localization of p16 and γH2AX. Adipose tissue samples for these evaluations had been stored at −80 °C and not subjected to repeated freeze and thaw. Also, samples from all subjects were treated in the same manner.

Additionally, we undertook experiments using complete adipose tissue explants to examine the effect of IH on senescence phenotype. Fresh subcutaneous abdominal adipose tissue samples obtained from the healthy individual was aliquoted and treated for 3, 5 and 7 days in NO or IH. After treatment, the tissue sample was fixed with 4% paraformaldehyde prior to SA-β-gal staining (described above). Visible difference in coloration of adipose tissue was used to identify increased SA-β-gal activity in adipose tissue.

### Statistical analysis

Data are presented as mean and standard error of the mean. In *in vitro* experiments the observation between any treatment and the control group were treated as paired observations as samples were derived from cells of the same lot and paired t-tests were used for pairwise significance. Primary comparison groups and direction for the outcome (increase/decrease) for each experiment were determined ‘a priori’ with significance level set at *P* < 0.05. Importantly, since our hypothesis reflects directionality, we chose to use one-tailed t-test. Other comparisons were secondary and as such considered to be an exploratory analysis. For example, in experiments evaluating the effects of drugs in attenuating detrimental effects of IH exposure, primary comparisons were defined as IH condition alone versus IH condition + treatment with hypothesis that treatment will reduce the levels of ROS/H2AX foci/β-gal positive cells. Cell grown in continuous NO served as an experimental control. Furthermore, considering the small sample sizes from *in-vitro* experiments, we would essentially have no power to detect “non-normality”. Therefore, we had decided beforehand to not test for the distribution to reduce the Type 1 error rate.

For human subjects, independent two-sample t-test was used to test the difference in the subject characteristics. Linear regression model adjusted for age and BMI was used to analyze the effect of OSA status and a given treatment on levels of γH2AX & p16 positive nuclei in human adipose tissue. *P*-values < 0.05 were considered statistically significant. All analysis were performed in JMP® Pro 10.0.0.

## Supplementary information

Supplementary Figures.

## Data Availability

The datasets generated and analyzed during the current study are available from the corresponding author on reasonable request.
