# Supplementary material for: Chronic Intermittent Hypoxia Triggers a Senescence-like Phenotype in Human White Preadipocytes
Source: Sci Rep. 2020 Apr 22;10:6846. doi: 10.1038/s41598-020-63761-7 (PMC7176724; doi:10.1038/s41598-020-63761-7)
Supplement: Supplementary file 1 — Supplementary Figures. [file 41598_2020_63761_MOESM1_ESM.pdf]

CHRONIC INTERMITTENT HYPOXIA TRIGGERS A SENESCENCE-LIKE PHENOTYPE IN HUMAN WHITE PREADIPOCYTES

Katarzyna Polonis[#], Christiane Becari[#], C. Anwar A. Chahal, Yuebo Zhang, Alina M. Allen, Todd A. Kellogg, Virend K. Somers, Prachi Singh*

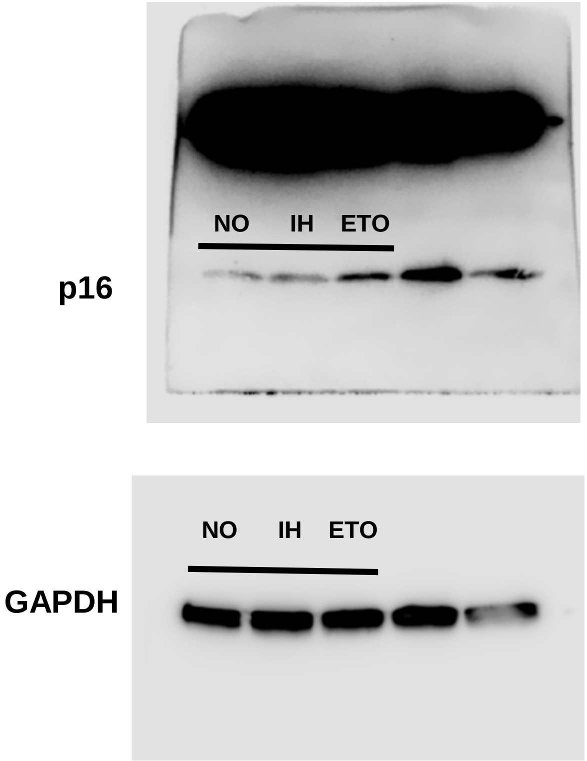

Figure 2 a

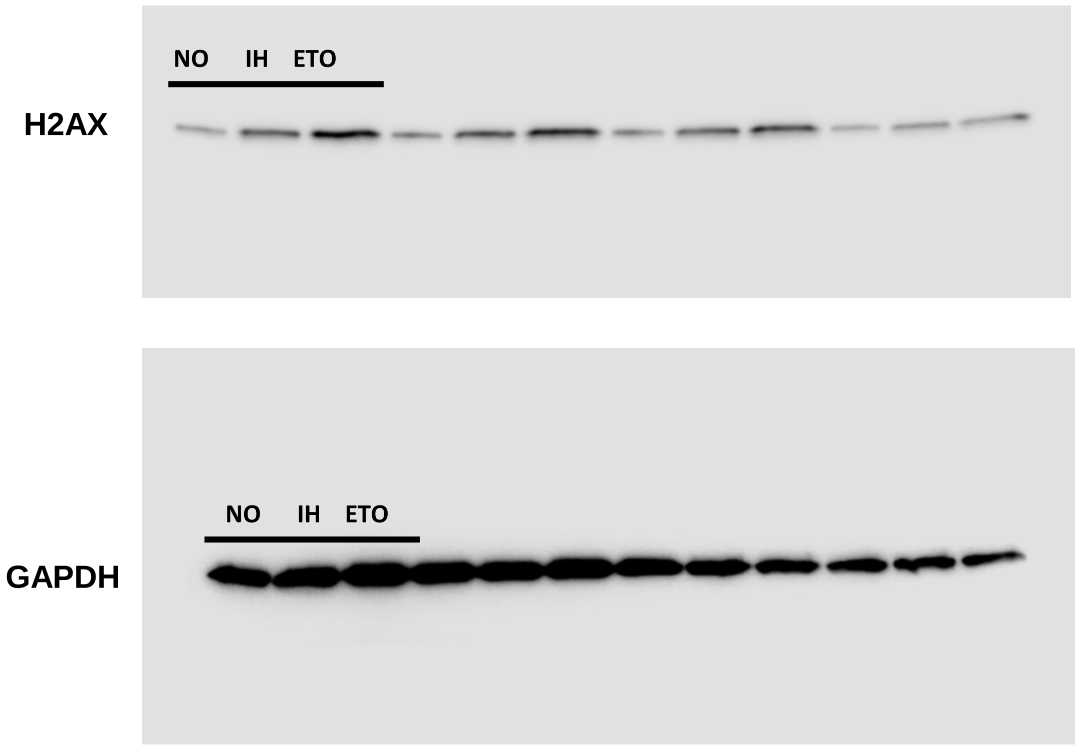

Figure 2 b

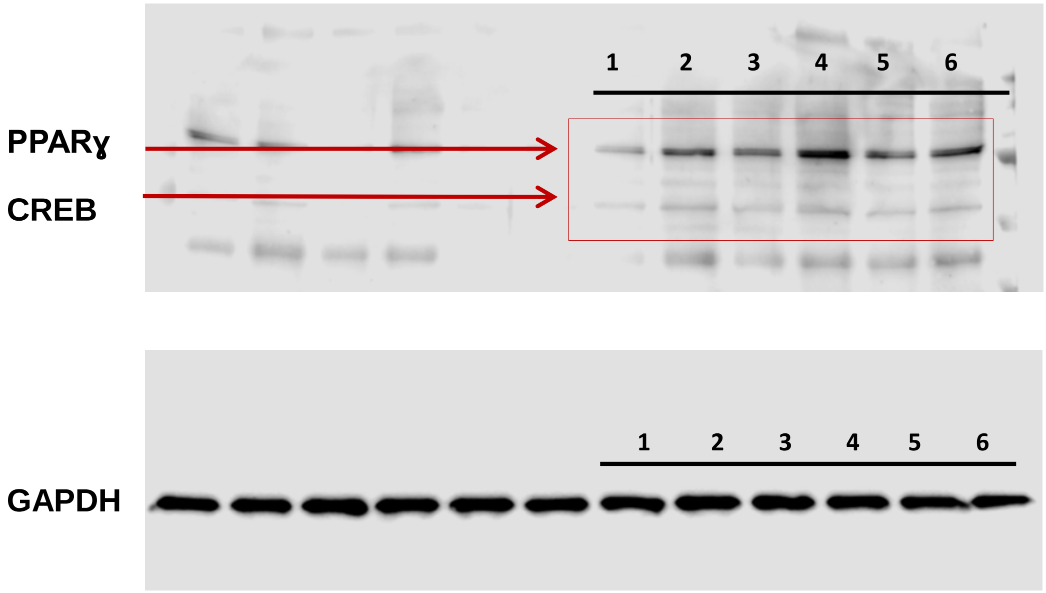

Figure 4 c and d

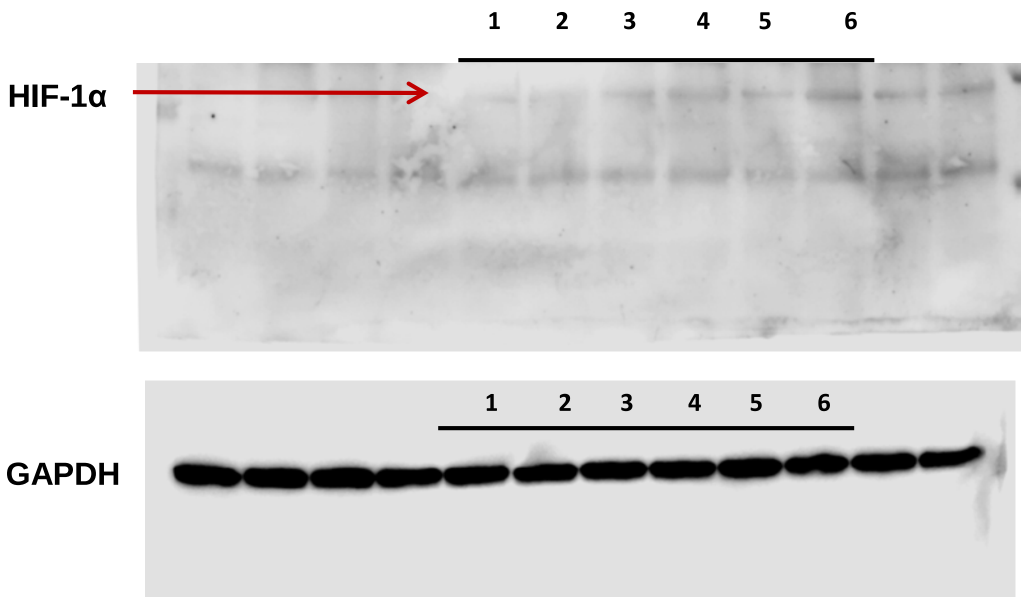

Figure 4 e
